# Lefamulin dosing optimization using population pharmacokinetic and pharmacokinetic/pharmacodynamic assessment in Chinese patients with community-acquired bacterial pneumonia

**DOI:** 10.3389/fphar.2024.1456741

**Published:** 2024-10-25

**Authors:** Xingchen Bian, Nanyang Li, Yi Li, Xu Zhu, Jicheng Yu, Yingying Hu, Haijing Yang, Qiong Wei, Xiaojie Wu, Jingjing Wang, Guoying Cao, Jufang Wu, Yang Wang, Jing Zhang

**Affiliations:** ^1^ Clinical Pharmacology Research Center, Huashan Hospital, Fudan University, Shanghai, China; ^2^ National Clinical Research Center for Aging and Medicine, Huashan Hospital, Fudan University, Shanghai, China; ^3^ Research Ward of Huashan Hospital, Fudan University, Shanghai, China; ^4^ Institute of Antibiotics, Huashan Hospital, Fudan University, Shanghai, China; ^5^ Key Laboratory of Clinical Pharmacology of Antibiotics, Shanghai, China; ^6^ Sumitomo Pharmaceuticals (Suzhou) Co., Ltd., Shanghai, China

**Keywords:** lefamulin, Chinese population, population pharmacokinetic, pharmacokinetic/pharmacodanamic, community-acquired bacterial pneumonia

## Abstract

**Purpose:**

Lefamulin is the first pleuromutilin antibiotic approved for the treatment of community-acquired bacterial pneumonia (CABP). However, the pharmacokinetic/pharmacodynamic (PK/PD) characteristics in Chinese CABP patients are not fully understood. This study aimed to evaluate its microbiological efficacy against *Streptococcus pneumoniae* and *Staphylococcus aureus* via PK/PD analysis.

**Methods:**

The population PK (PopPK) model, established with foreign data was validated using data from Chinese CABP patients. PK/PD analysis was conducted for the intravenous administration of 150 mg q12 h for 1-h, 1.5-h and extended 2-h infusion. Oral administrations of 600 mg q12 h were assessed, considering original and higher plasma protein binding.

**Results:**

Lefamulin displayed similar PK characteristics in both Chinese and Western populations. The PopPK model effectively predicted lefamulin concentrations in Chinese CABP patients. Under the dosage regimen of 150 mg q12 h via intravenous infusion for 1/1.5/2 h, the probability of target attainments reached 98% at the minimum inhibitory concentration for both 90% *S. pneumoniae* and *S. aureus,* considering both original and higher protein binding rates. It is advisable to extend the infusion duration from 1/1.5 h–2 h to minimize the risk of adverse effects at the infusion site. Regardless of fasted or fed conditions, the PTAs for 600 mg q12 h lefamulin via oral administration proved comparable to those for intravenous administration.

**Conclusion:**

This study proved that intravenous and oral administrations of lefamulin can reach preclinical PK/PD targets of *S. pneumoniae* and *S. aureus*. These findings support the optimal use of lefamulin for the safe and effective treatment of Chinese CABP patients.

## Introduction

Lefamulin, the first systemic pleuromutilin antibiotic for treating community-acquired bacterial pneumonia (CABP) (Chahine and Sucher, 2020; Zhanel et al., 2021), has been approved by the U.S. Food and Drug Administration (FDA) in 2019 (3), the European Medicines Agency (EMA) in 2020, and the China National Medical Products Administration (NMPA) in 2023, respectively. It exhibits a broad spectrum of antimicrobial activity against respiratory pathogens, including *Staphylococcus aureus* and *Streptococcus pneumoniae* regardless of resistance phenotype or genotype (Zhanel et al., 2021; Wu et al., 2020). Recommended dosing regimens involve either a 1/1.5-h intravenous infusion of 150 mg q12 h, oral administration of 600 mg q12 h, or a sequential combination of intravenous and oral administration (Xenleta, 2019; Covvey and Guarascio, 2022; Lefamulin Acetate Concentrated Solution for Injection, 2023). Clinical development of lefamulin in China commenced in 2019, with completed studies including a clinical trial in healthy subjects (Hu et al., 2023) and an efficacy comparison study with moxifloxacin in CABP patients. The pharmacokinetic similarities between Chinese and Western populations, as well as the pharmacokinetic/pharmacodynamic (PK/PD) relationships in Chinese patients, remain unclear. Bhavnani et al. (2019) evaluated lefamulin PK/PD based on pharmacokinetic data from healthy volunteers. However, the exposure of lefamulin in patients with CABP was higher than that in healthy volunteers which will affect the PK/PD evaluation. From the clinical pharmacology report of FDA, the mean AUC_0-24_ and C_max_ in CABP patients was approximately 1.73- and 1.3-fold greater compared to adults without pneumonia following the therapeutic IV and PO dosing regimens on Day 1 (9). This study compared PK similarities of lefamulin in Chinese and Western populations and conducted PK/PD analysis to assess its microbiological efficacy against *S. pneumoniae* (*S. pneumoniae*) and *S. aureus* (*S. aureus*) with both intravenous and oral administrations in patients with CABP. Additionally, the lefamulin package insert notes adverse reactions related to intravenous formulations, specifically administration site reactions, with an incidence of 7% (19/273) (Xenleta, 2019). To address above situation, this study proposed extending the infusion time from 1/1.5 h–2 h and evaluating the microbiological efficacy under extended infusion through PK/PD analysis.

The population pharmacokinetic (PopPK) data from Chinese CABP patients was used as an external validation dataset to assess the applicability of the PopPK model established based on foreign data in predicting lefamulin concentrations in Chinese CABP patients. Considering that the drug exposure at the infection site is more relevant to microbiological efficacy (Levison and Levison, 2009), this study simultaneously simulated the lefamulin exposure in plasma and epithelial lining fluid (ELF) with intravenous and oral dosage (fasted vs. fed) based on the validated PopPK model (Zhang et al., 2019). PK/PD assessment was conducted using pharmacodynamic data of *S. pneumoniae* (*S. pneumoniae*) and *S. aureus* (*S. aureus*) from China to evaluate the impact of infusion time and food on exposure, probability of target attainment (PTA), and cumulative fraction of response (CFR).

Plasma protein binding plays a key role in drug therapy, affecting PK and PD. The plasma protein binding parameters of lefamulin used in the established PopPK model were derived from an *in vitro* study, and the binding rate was 73%–88% (Nabriva Therapeutics, 2019) (original plasma protein binding rate). During the review process, the FDA noted that another *in vitro* plasma protein binding study resulted in a higher binding rate, with a binding rate of 86%–97% (Nabriva Therapeutics, 2019) (higher plasma protein binding rate). When the FDA conducted a PK/PD compliance probability analysis, it chose to re-estimate the parameters of the existing PopPK model (without changing the structure of the PopPK model) using a higher binding rate (Nabriva Therapeutics, 2019). This study assessed the effects of original and higher plasma protein binding rates on PTA, and anticipated microbial efficacy of lefamulin in Chinese CABP patients. The findings could offer guidance for the clinical application of lefamulin upon its introduction in China, serving as a foundation for practical use.

## Methods

### PopPK dataset

The PopPK dataset comprises subjects with at least one assessable drug concentration datum. The dataset encompasses a range of information, including plasma drug concentrations, dosing, PK sampling, demographic characteristics, clinical laboratory results, and study phase. This study incorporated data from the Chinese Phase III clinical trial (BC-3781-S301), with a total of 217 plasma drug concentration entries from 33 subjects. Table 1 summarizes the number of subjects, concentrations stratified by administration route, and data below the lower limit of quantitation. The concentrations of lefamulin were determined via liquid chromatography-tandem mass spectrometry after protein precipitation treatment.

**TABLE 1:** A summary of the number of plasma concentrations in the database.

Administration route*	Number	Dose (mg)	Number of initial concentrations	Number of rejected concentrations		Number of patients in final PopPK model (%)	Number of concentrations in final PopPK model (%)
				BQL before administration (%)	BQL after administration (%)		
IV	33	150	166	33 (19.88%)	0	33 (100.00%)	133 (80.12%)
PO	18	600	51	0	1 (1.96%)	18 (100.00%)	50 (98.04%)
ALL	33	150/600	217	33 (15.21%)	1 (0.46%)	33 (100.00%)	183 (84.33%)

* IV, intravenous administration; PO, oral administration; ALL, intravenous administration + oral administration, BQL, below the limit of quantitation.

### PopPK model of lefamulin

**FIGURE 1:**
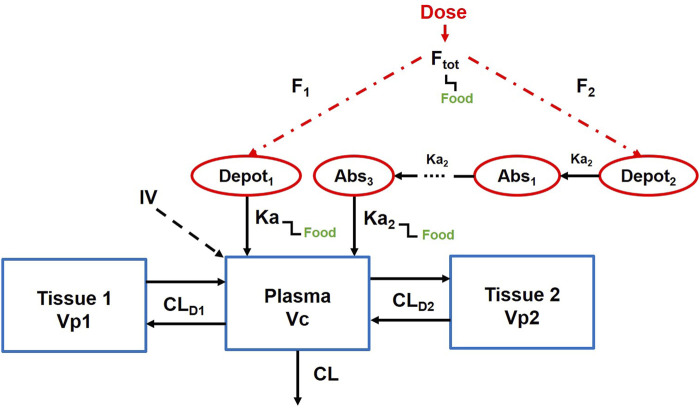
Population pharmacokinetic model of lefamulin.

Figure 1 illustrates the established PopPK model (Nabriva Therapeutics, 2019). The model consists of one central compartment and two peripheral compartments with parallel rapid and delayed absorption pathways. Additionally, it assumes linear elimination of the drug from the central compartment. The model employs an exponential model to characterize inter-individual variability and utilizes a mixed-effects model incorporating additive and proportional components to describe residuals. Covariates integrated into the model encompass albumin levels, body weight, and study phase.

CL, clearance, CL_D1,_ distributional clearance to the first peripheral compartment, CL_D2,_ distributional clearance to the second peripheral compartment, V_c_, volume of distribution of the central compartment, V_p1_, volume of distribution for the first peripheral compartment, V_p2_, volume of distribution for the second peripheral compartment, K_a_, absorption rate constant through the immediate process, K_a2_, absorption rate constant through the delayed process, F_tot_, total oral bioavailability.

The fixed-effect parameters in the model encompass clearance (CL), distributional clearance to the first peripheral compartment (CL_D1_), distributional clearance to the second peripheral compartment (CL_D2_), volume of distribution of the central compartment (V_c_), volume of distribution for the first peripheral compartment (V_p1_), volume of distribution for the second peripheral compartment (V_p2_), absorption rate constant through the immediate process (K_a_), absorption rate constant through the delayed process (K_a2_), total oral bioavailability (F_tot_), fraction of dose absorbed through slow absorption (FS), minimum fraction unbound of the free drug (*f*_*u, min*_), maximum fraction unbound of the free drug (*f*_*u, max*_), concentration at which the fraction unbound is half-maximal (*f*_*u50*_), and lag time (ALAG). These fixed parameters (θ_x_) and their corresponding random-effect parameters (η_x_) are described by the following formula (Equation 1).

$$CL\;\left(L/h\right):CL=\left[1+\theta_{20}\times \left(ALB-4.1\right)\right]\times CLPHASE\times \theta_{1}\times \exp\left(\eta_{1}\right)$$ (1)

When PHASE = 3, CLPHASE = 1, when PHASE = 2, CLPHASE=(1+θ_21_), when PHASE = 1, CLPHASE=(1+θ_22_). ALB represents the individual plasma albumin level (g/dL), serving as a covariate influencing CL.

$$V_{c}\;\left(L\right)༚Vc=\theta_{2}\times \exp\left(\eta_{2}\right)$$ (2)

$$CL_{D1}\;\left(L/h\right)༚Q_{1}=Q_{1}PHASE\times \exp\left(\eta_{3}\right)$$ (3)

When PHASE = 3, Q_1_PHASE = 1, when PHASE = 2, Q_1_PHASE=(1+θ_23_), when PHASE = 1, Q_1_PHASE=(1+θ_24_) (Equations 2, 3).

$$V_{p1}\;\left(L\right)༚V_{p1}=V_{p1}PHASE\times \left(1+\theta_{27}\times \left(WTKG-78\right)\right)\times \exp\left(\eta_{4}\right)$$ (4)

When PHASE = 3, V_p1_PHASE = 1, when PHASE = 2, V_p1_PHASE=(1+θ_25_), when PHASE = 1, V_p1_PHASE = (1+θ_26_). WTKG is the individual body weight, serving as a covariate influencing V_p1_ (Equation 4). The CL_D2_ and V_p2_ were described as Equations 5, 6, respectively.

$$CL_{D2}\;\left(L/h\right)༚Q_{2}=\theta_{5}\times \exp\left(\eta_{5}\right)$$ (5)

$$V_{p2}\;\left(L\right)༚V_{p2}=\theta_{6}\times \exp\left(\eta_{5}\right)\times \exp\left(\eta_{6}\right)$$ (6)

$$K_{a}\;\left(1/h\right)༚Ka=\theta_{7}\times \text{FAST}+\theta_{7}\times \theta_{19}\times FEDD\times d\exp\left(\eta_{7}\right)$$ (7)

In the fasted state, FAST = 1, FEDD = 0. In the fed state, FAST = 0, FEDD = 1 (applicable to Equations 7–9).

$$K_{a2}\;\left(1/h\right)༚Ka_{2}=\theta_{8}\times FAST+\theta_{8}\times \theta_{17}\times FEDD\times d\exp\left(\eta_{8}\right)$$ (8)

$$F_{\text{tot}}༚BIO=\theta_{9}\times FAST+\theta_{9}\times \theta_{18}\times FEDD$$ (9)

When BIO>0, $FPO=\mathrm{LOG}\left(BIO/\left(1-BIO\right)\right)$ , when BIO = 0, FPO = 0 (Equation 10).

$$F_{tot}=\exp\left(FPO+\eta_{9}\right)/\left(1+\exp\left(FPO+\eta_{9}\right)\right)$$ (10)

$$F_{1},F_{2}༚FSA=\mathrm{LOG}\left(\theta_{10}/\left(1-\theta_{10}\right)\right)$$ (11)

$$FS=\exp\left(FSA+\eta_{10}\right)/\left(1+\exp\left(FSA+\eta_{10}\right)\right)$$ (12)

The fraction of lefamulin in the delayed absorption part, FS, can be used to calculate the bioavailability of immediate absorption (F1) and the bioavailability of delayed absorption (F2) (Equations 11, 12). The calculation formulas are as follows (Equations 13, 14).

$$F_{1}=F_{tot}\times \left(1-FS\right)$$ (13)

$$F_{2}=F_{tot}\times FS$$ (14)

When administered intravenously, the default bioavailability is set to 1.

$$ALAG\;\left(h\right)༚\text{ALAG}=\theta_{11}$$ (15)

Regarding the lag time, the lag time for immediate absorption and the lag time for delayed absorption are assumed to be equal in duration (Equation 15).

The calculation formulas for the free fraction of lefamulin, which follows a Sigmoid E_max_ model, are as follows (Equations 16–19).

$$f_{u}=f_{u,\min}+f_{u,\max}*C/\left(f_{u50}+C\right)$$ (16)

$$f_{u,\min}:f_{u,\min}=\theta_{12}$$ (17)

$$f_{u,\max}:f_{u,\min}=\theta_{13}$$ (18)

$$f_{u50}༚f_{u50}=\theta_{14}$$ (19)

The concentrations of lefamulin in plasma or ELF were calculated according to the following formulas (Equation 20).

$$Y=IPRED+IPRED*\varepsilon_{1}*PLASMA+\varepsilon_{2}*PLASMA+IPRED*\varepsilon_{3}*ELF$$ (20)

In central compartment, PLASMA = 1, ELF = 0 while in ELF compartment, PLASMA = 0, ELF = 1. IPRED refers to the individual predicted concentrations.

External validation was performed using the Bayesian posterior method of the Nonlinear Mixed Effects Model (NONMEM) to estimate individual predicted concentrations. Model bias and precision were assessed by comparing the model-predicted values with observed values. This assessment included the calculation of mean prediction error (MPE), mean absolute prediction error (MAPE), and root mean square prediction error (RMSE) based on the predicted and observed concentrations of lefamulin. Additionally, MPE%, MAPE%, and RMSE% were utilized to evaluate the accuracy and precision of the model. The predictive performance was further evaluated by goodness of fit (GOF), visual predictive check (VPC), and normalized prediction distribution errors (NPDE).

### PopPK model integrating ELF distribution compartment

Based on the lefamulin concentrations in plasma and ELF obtained from foreign clinical studies, a PopPK model incorporating an ELF distribution compartment has been established.

**FIGURE 2:**
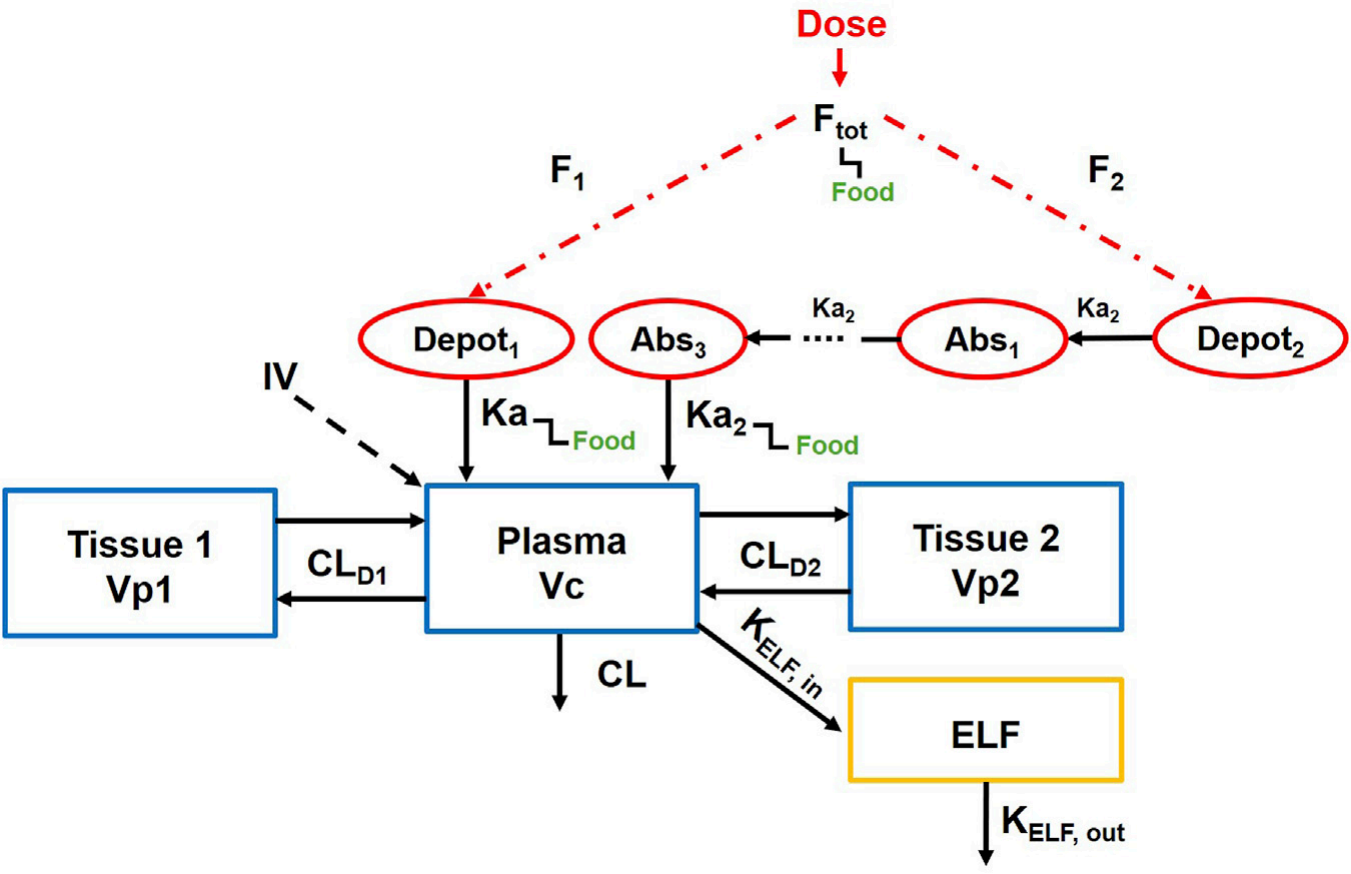
Population pharmacokinetic model of lefamulin integrating epithelial lining fluid compartment.

When the original protein binding rate was used, ELF, as an effect compartment, was linked to the central compartment of the PopPK model. The fixed-effect parameters, including the distribution rate constant from the central compartment to the ELF compartment (K_ELF,in_) and the elimination rate constant from the ELF compartment (K_EFL,out_) for lefamulin, are derived from the previously established model. The model diagram and the parameter values are shown in Figure 2; Supplementary Table S1.

ELF distribution model from the FDA review report was adopted when a higher protein binding rate was utilized.

$$C_{ELF}=LPR\left(\frac{1mg}{L}\right)\times {\left[C_{p}\left(t\right)\times 0.0379\right]}^{power}$$ (21)

LPR (1 mg/L) represents the penetration rate from plasma (lefamulin concentration of 1 mg/L) to ELF, Cp(t) is plasma drug concentration. The exponent power induces changes in permeability as the plasma drug concentration varies. The model parameters are shown in Supplementary Table S2.

### Pharmacokinetic simulation

The simulation dataset was from a phase III study involving 125 Chinese CABP patients (baseline body weight and albumin levels), a covariate dataset for 5000 CABP patients was generated through a resampling approach. Simulations were conducted for lefamulin 150 mg q12 h intravenous infusion over 1 h, 1.5 h and 2 h, as well as 600 mg q12 h oral administration under fasted or fed conditions. During simulations, numerical integration was employed to calculate the plasma free drug exposure from 0 to 24 h (*f*AUC_0–24h, plasma_) and the ELF drug exposure (AUC_0–24h, ELF_) at steady state (Day 3).

### Pharmacokinetic/pharmacodynamic assessment

From the Chinese lefamulin pharmacodynamic study, the minimum inhibitory concentration (MIC) distributions of *S. pneumoniae* and *S. aureus* isolated between 2017 and 2019 are shown in Supplementary Table S3, (Wu et al., 2020).

The PK/PD indice of lefamulin is *f*AUC_0–24h_/MIC (Bhavnani et al., 2019; Rodvold, 2019). In the mouse *in vivo* pneumonia model, the median targets of free drug in plasma and total drug in ELF were demonstrated in Supplementary Table S4 (Wicha et al., 2019a). Monte Carlo simulation was conducted to estimate the probability target attainment (PTA) and cumulative fraction of response (CFR) of lefamulin against *S. pneumoniae* and *S. aureus*. Dosage regimens with PTA and CFR >90% were considered as microbiologically effective.

## Results

### PopPK dataset

The descriptive statistics for continuous and categorical covariates of subjects in the modeling dataset and the validation dataset are presented in Supplementary Table S5. In the Chinese Phase III trial, the values of continuous covariates for subjects (including age, height, weight, body surface area, creatinine clearance and albumin) fall within the range observed in foreign subjects, showing similarity with the values observed in the foreign Phase III trial. For categorical covariates (including gender and race), in the Chinese Phase III study, the proportion of male subjects is higher than in the foreign study, with percentages of 81.82% for males in the Chinese study, compared to 57.9% males in the foreign Phase III study. The foreign Phase III study includes subjects from different racial groups, such as White, Black, and Asian populations, with Asians comprising 11.3%. In contrast, the Chinese Phase III study only includes Asian subjects (all Chinese). The final covariates included in the foreign PopPK model are albumin, weight, and study phase.

**FIGURE 3:**
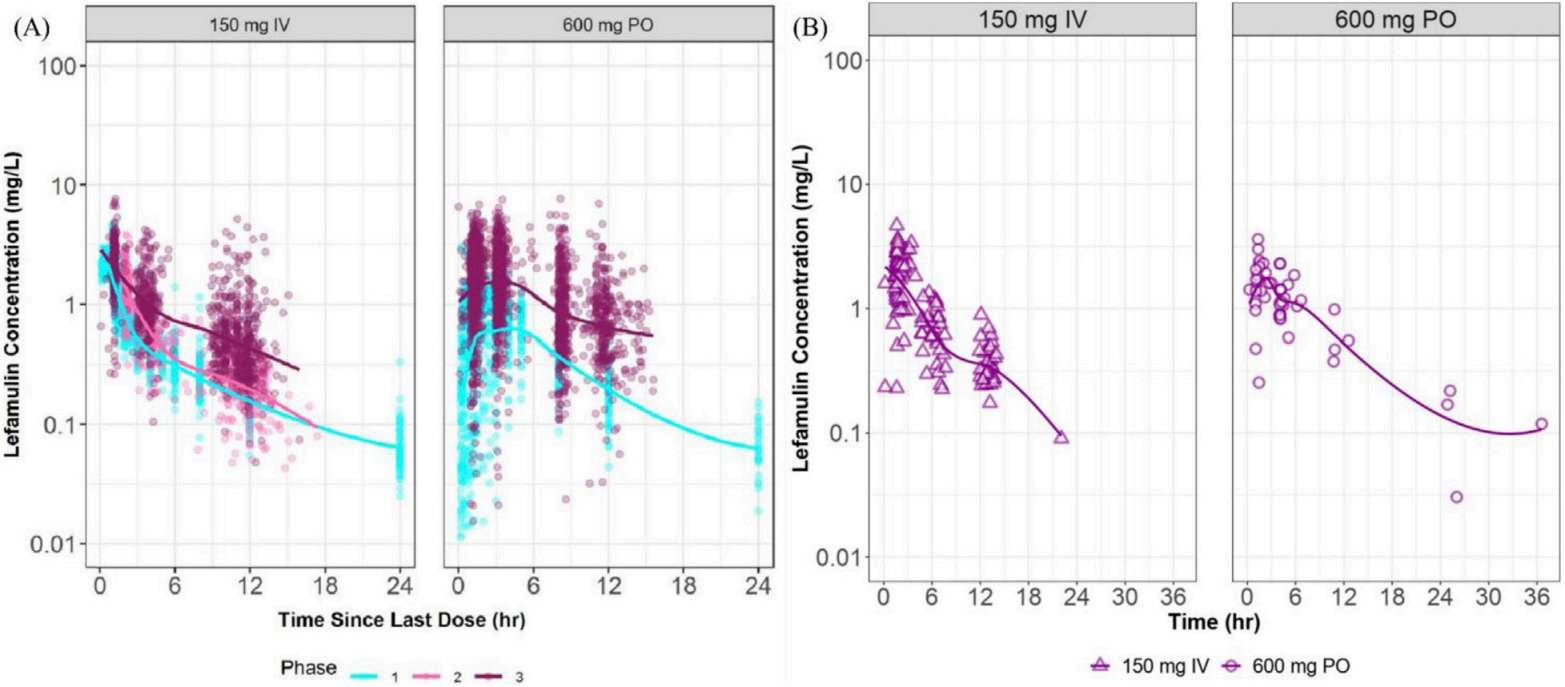
Plasma concentration vs. time curves at steady state in foreign and Chinese studies. **(A)** presents semi-logarithmic drug concentration-time curves of lefamulin from foreign clinical studies stratified by administration route and study phase, with different colors representing distinct study phases. **(B)** illustrates semi-logarithmic drug concentration-time curves of lefamulin from the Chinese Phase III clinical study, stratified by administration route. IV, intravenous administration, PO, oral administration.

The distribution characteristics of the concentration-time curves for intravenous (150 mg) and oral (600 mg) administration in the Chinese Phase III study closely resemble the features of corresponding curves observed in foreign studies (Figure 3). The concentration distribution ranges are similar across different administration routes.

### Predictive error evaluation

Based on the predictions and observed values of lefamulin concentrations in Chinese patients with CABP, the model prediction errors are summarized in Table 2. The overall MPE is −0.01, with a 95% confidence interval (CI) of −0.08 to 0.06. The MPE% is 10.90%. When administered intravenously and orally, the MPE is 0.02 and −0.07, and the MPE% is 15.60% and −1.70%, respectively. These results indicate that the overall model predictions are close to the observed values, with small relative errors (MPE% ≤ 20%).

**TABLE 2:** Prediction errors for different administration routes.

Administration route	MPE [95%CI]	MPE% (%)	MAPE [95%CI]	MAPE% (%)	RMSE [95%CI]	RMSE% (%)	F20 (%)	F30 (%)
IV	0.02 [−0.07, 0.11]	15.60	0.30 [0.23, 0.37]	30.00	0.50 [0.45, 0.57]	72.70	61.65	76.69
PO	−0.07 [-0.14, 0]	−1.70	0.19 [0.14, 0.24]	15.30	0.24 [0.2, 0.3]	18.90	70.00	90.00
All	−0.01 [-0.08, 0.06]	10.90	0.27 [0.22, 0.32]	25.90	0.45 [0.41, 0.5]	62.70	63.93	80.33

IV, intravenous administration; PO, oral administration, All, intravenous administration + oral administration, MPE, mean prediction error; MAPE, mean absolute prediction error; RMSE, root mean square prediction error.

The overall MAPE and its relative error MAPE% are 0.27% and 25.90%, respectively. The overall RMSE and RMSE% are 0.45% and 62.70%, respectively. For intravenous and oral administration, the MAPE is 0.30 and 0.19, the MAPE% is 30.0% and 15.30%, the RMSE is 0.50 and 0.24, and the RMSE% is 72.70% and 18.90%, respectively. For composite indicators F_20_ and F_30_, which simultaneously represent prediction accuracy and precision, the overall F_20_ and F_30_ are 63.93% and 80.33%, respectively. For intravenous administration, F_20_ and F_30_ are 61.65% and 76.69%, while for oral administration, F_20_ and F_30_ are 70.00% and 90.00%, respectively. These findings suggest that the model adequately describes the validation dataset. (MAPE% ≤ 30%, F_20_ ≥ 35%, F_30_ ≥ 50%).

### Visualization assessment of model predictive performance

**FIGURE 4:**
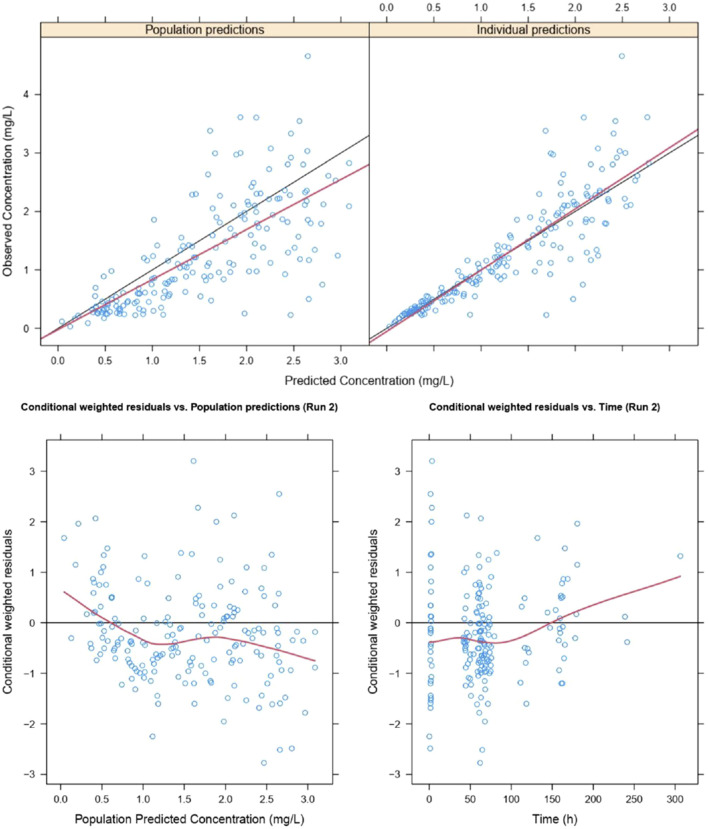
Goodness of fit of lefamulin final PopPK model. The blue dots are observed values, the black lines are diagonal lines, the red lines are loess regression curves.

**FIGURE 5:**
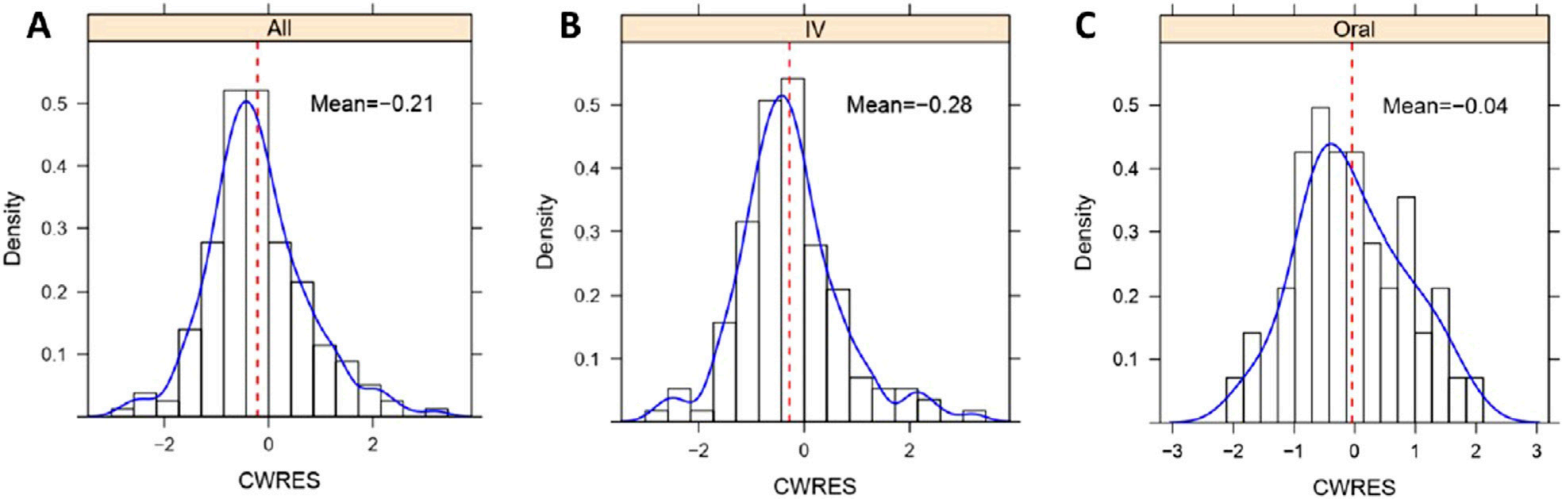
Histogram of CWRES distribution of lefamulin final PopPK model. **(A)** CWRES distribution for intravenous and oral administrations. **(B)** CWRES distribution for intravenous administrations. **(C)** CWRES distribution for oral administrations. IV, intravenous administration, Oral, oral administration, All, intravenous administration + oral administration.

The goodness of fit and conditional weighted residuals (CWRES) distribution for the final lefamulin PopPK model are presented in Figures 4, 5, respectively. As shown in Figure 4, the overall GOF indicates good consistency between individual predicted and observed values, with the trend line coinciding with the diagonal line. This suggests that the lefamulin PopPK model fits well with the concentration observations of Chinese CABP patients. The majority of the overall CWRES are distributed within ±2, and they are relatively evenly distributed around 0. The less accurate predictions at later timepoints may due to the sparse concentration datapoints.

Similarly, as illustrated in Supplementary Figure S1, the GOF indicates a good fit of the model for both the intravenous and oral administrations. In the histogram of CWRES distribution (Figure 5), the overall CWRES is distributed relatively evenly around 0 (Figure 5A), resembling a normal distribution. This suggests that the model has a minimal bias in predicting lefamulin plasma concentrations, indicating a well-fitted model. The CWRES distribution histograms for different administration routes exhibit a similar pattern to the overall distribution (Figures 5B, C).

**FIGURE 6:**
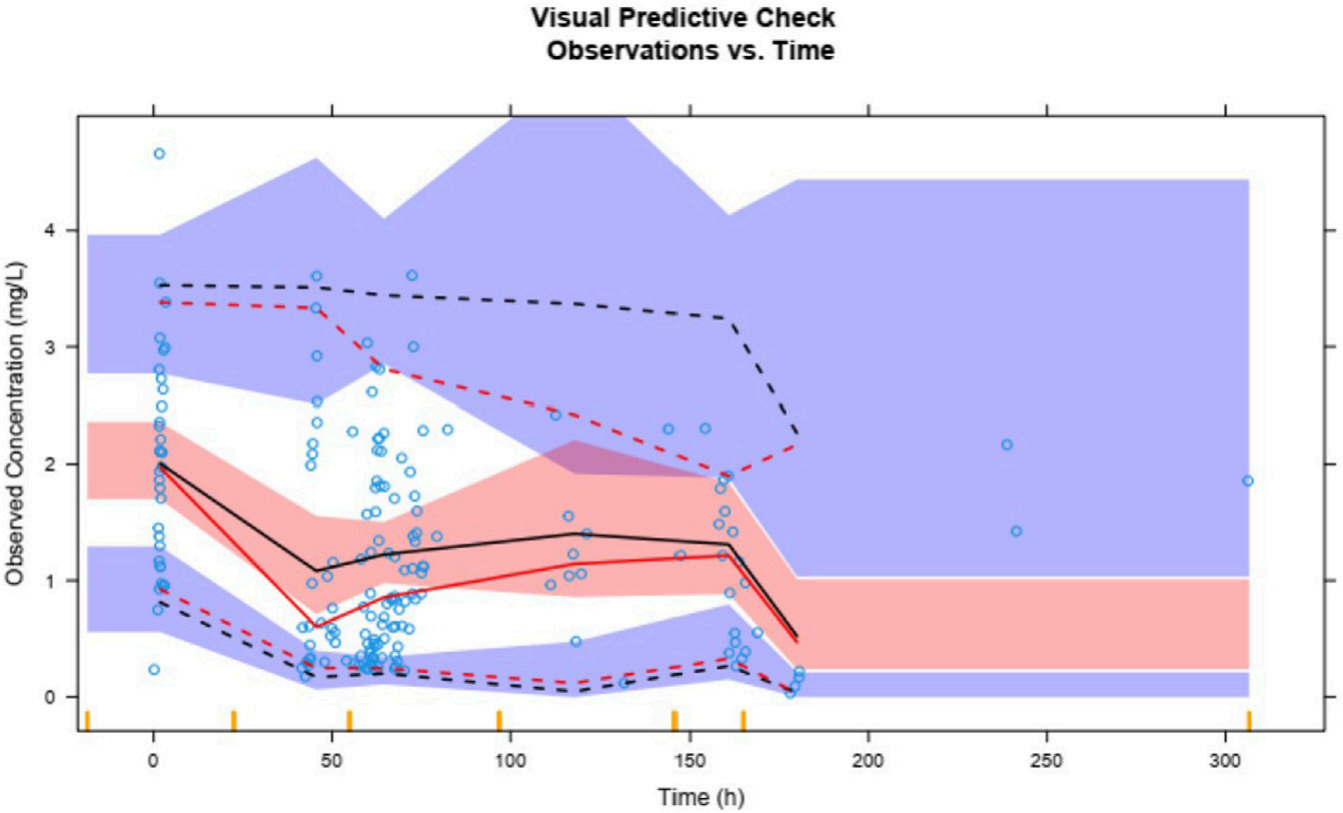
Visual predictive check of lefamulin final PopPK model.

Plasma concentrations of lefamulin from 1,000 simulations show that the 95th, 50th, and fifth percentiles of observed plasma concentrations are generally within the 90% prediction intervals for the corresponding percentiles (Figure 6). The VPC results for different administration routes are similar to the overall VPC results (Supplementary Figure S2), indicating that the model exhibits good predictive performance.

The solid red line represents the 50th percentile of observed values, with the dashed red lines from top to bottom indicating the 95th and fifth percentiles of observed values. The solid black line represents the 50th percentile of predicted values, with dashed black lines from top to bottom indicating the 95th and fifth percentiles of predicted values. The blue and red shaded areas represent the 90% confidence intervals for the corresponding predicted percentiles.

The following statistical tests were conducted based on the NPDE: t-test, Fisher test, and Shapiro-Wilks (SW) test. These tests aimed to assess whether the distribution of NPDE significantly differs from a standard normal distribution. As shown in Table 3, the *P*-values for the overall and intravenous administration in the t-test, Fisher test, and SW test are all less than 0.05. This suggests a significant difference between the distribution of NPDE for the overall dataset and intravenous administration compared to a standard normal distribution. However, for oral administration, the *P*-values for the t-test and SW test are greater than 0.05, indicating relatively better predictive performance compared to intravenous administration.

**TABLE 3:** Normalized Prediction Distribution Errors of the final lefamulin PopPK model.

	N	Mean	Variance	Skewness	Kurtosis	t-test	Fisher test	SW test	Overall test
						*P*-value			
IV	133	−0.239	0.757	0.579	1.49	0.00193	0.0341	0.00122	0.00366
PO	50	0.0862	0.609	−0.351	0.226	**0.438**	0.0278	**0.658**	**0.0833**
All	183	−0.15	0.734	0.325	0.938	0.0189	0.00561	0.0229	0.0168

Test results with *P*-values greater than 0.05 are presented in bold font which indicating a better prediction ability of the model for oral administration than intravenous administration. IV, intravenous administration; PO, oral administration, All, intravenous administration + oral administration.

**FIGURE 7:**
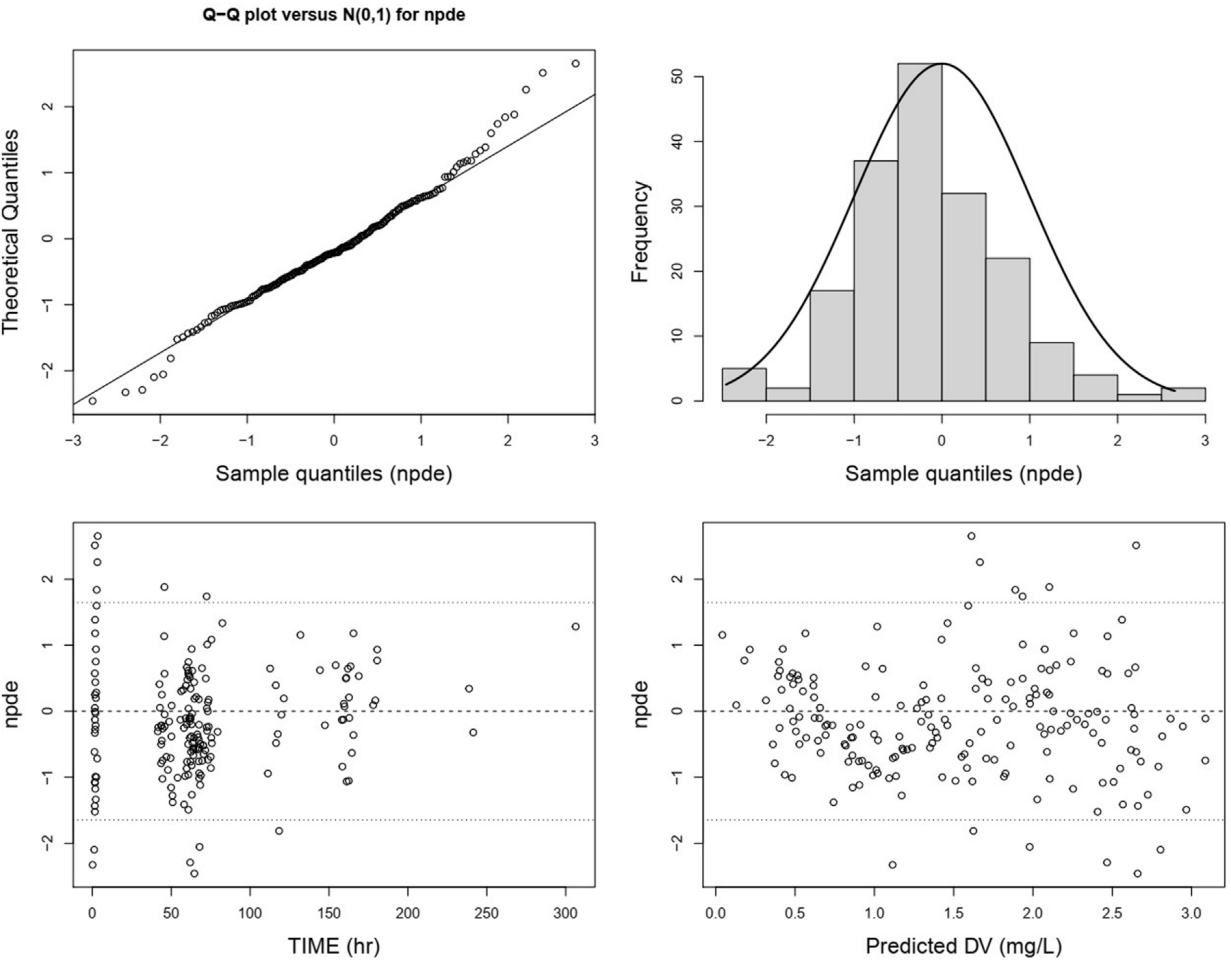
Normalized prediction distribution errors of the lefamulin final PopPK model. From the upper left to the lower right are the NPDE-standard normal distribution QQ plot, NPDE histogram, NPDE versus time plot, and NPDE versus predicted concentration plot.

The QQ plot and histogram of the overall NPDE show that the distribution is close to a standard normal distribution. In the NPDE vs. time and predicted concentration plot, NPDE is evenly distributed around the *X*-axis without exhibiting trend-related shifts (Figure 7). However, there are slight differences in the QQ plot and histogram of NPDE for different administration routes. The QQ plot for intravenous administration suggests some deviation in the distribution of NPDE from a standard normal distribution, and the histogram shows a leftward skew (Supplementary Figure S3A). In contrast, the QQ plot for oral administration indicates a distribution that closely follows a standard normal distribution (Supplementary Figure S3B).

### Exposure comparisons under different plasma protein binding

The plasma-free drug and ELF drug exposure achieved with each dosage regimen are shown in Supplementary Figure S4; Supplementary Table S6. The varying infusion times did not impact lefamulin exposure in plasma and ELF. ELF drug exposure levels were comparable under original and higher plasma protein binding rates. While plasma-free drug exposure levels were relatively lower under high protein binding rates. Lefamulin exposure in plasma and ELF achieved via 600 mg q12 h oral administration were similar to those achieved under 150 mg q12 h intravenous administration. In the fed state, the drug exposures decreased by 9%–42% compared to the fasted state.

### Probability of target attainment

Based on original and higher plasma protein binding rates, PK/PD analysis was performed based on the lefamulin exposure at steady state with the dosing regimen of 150 mg q12 h infused over 1/1.5/2 h, as well as the oral administration of 600 mg under fasted or fed conditions.

**FIGURE 8:**
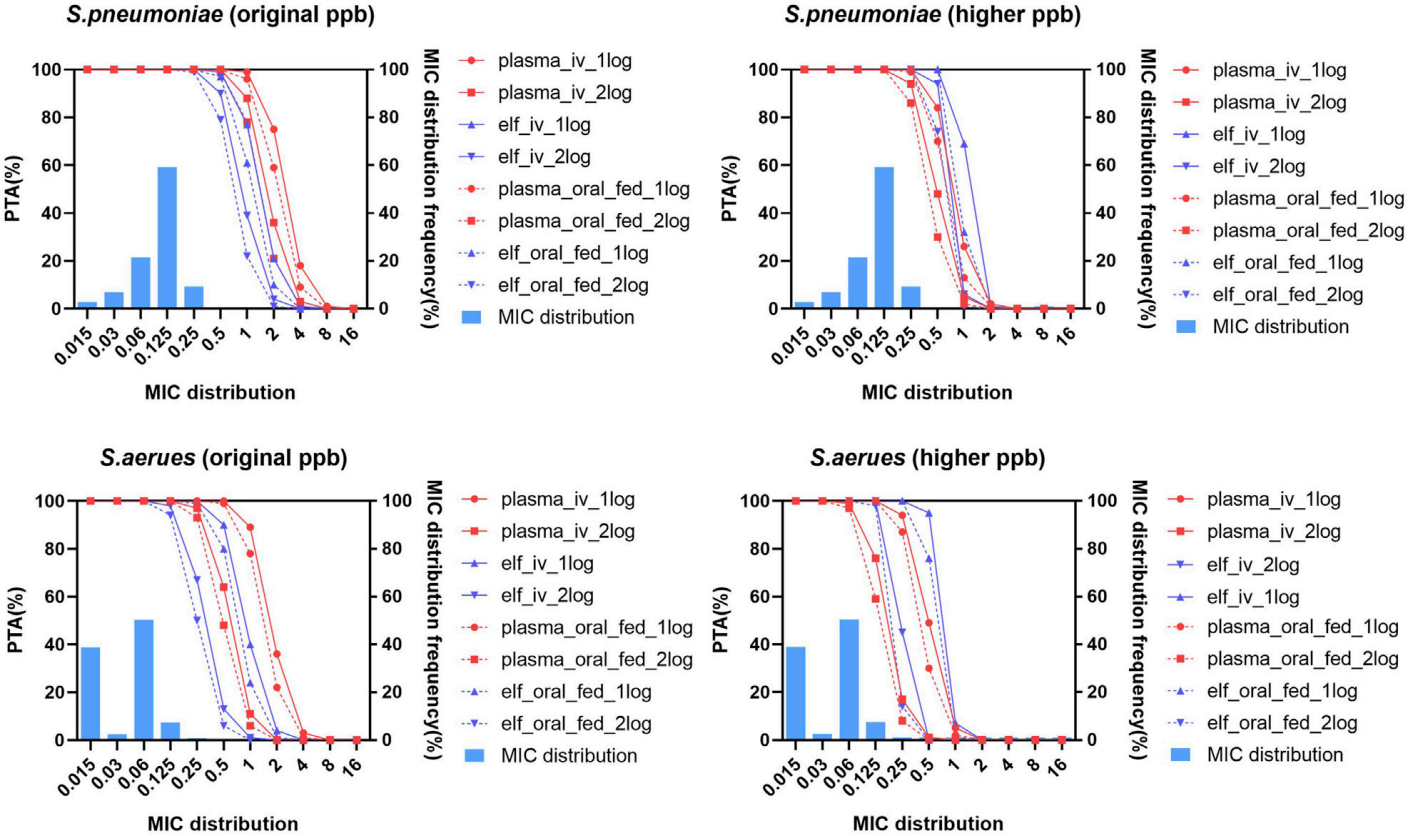
Probability of target attainment and MIC distribution for *Streptococcus pneumoniae* and *Staphylococcus aureus* in plasma and epithelial lining fluid with original and higher plasma protein binding after lefamulin intravenous and oral administration. Lefamulin was administered at 150 mg q12 h via a 1/1.5/2 h infusion or as an oral dose of 600 mg under fasted or fed conditions. PK/PD targets corresponding to 1-log_10_/2-log_10_ CFU/mL bacteria reductions were utilized. Original ppb refers to the diluted plasma protein binding, higher ppb refers to the non-diluted plasma protein binding rate. PTA, probability of target attainment, elf, epithelial lining fluid, MIC, minimum inhibitory concentration, *S. pneumoniae*, *Streptococcus pneumoniae*, *S. aureus*, *Staphylococcus aureus*.

The PTA achieved with oral dose of 600 mg under fasted condition (not shown in Figure 8) was highly similar to those with 150 mg q12 h via a 1/1.5/2 h infusion.When the pharmacodynamic tartet achieving 1-log_10_ CFU/mL was utilized in PK/PD analysis, all the intravenous and oral administrations can reach PTA of 98% at MIC_90_ of *S. pneumonia* and *S. aureus* with both original and higher protein binding rates. When the pharmacodynamic tartet of 2-log_10_ CFU/mL was utilized, all the intravenous and oral administrations under fasted condition can reach PTA of 94% at MIC_90_ of *S. pneumonia* and *S. aureus* with both original and higher protein binding rates (Supplementary Table S7; Figure 8).

### Pharmacokinetic/pharmacodynamic breakpoints

The PK/PD breakpoints of lefamulin with the recommended dosage regimens were almost all higher than MIC_50_/MIC_90_ of *S*. *pneumoniae* and *S. aureus* indicating that these two bacteria species are generally susceptible to lefamulin (Supplementary Table S8).

### Cumulative fraction of response

The cumulative response percentages of different bacteria to lefamulin at different protein binding rates are shown in Table 4. Under both lower and higher protein binding rates, the dosing regimen of lefamulin 150 mg q12 h administered via intravenous infusion over 1/1.5/2 h resulted in cumulative response percentages exceeding 95%. This suggests that the proposed regimen achieves a favorable microbiological efficacy in the treatment of community-acquired pneumonia caused by *S. pneumoniae* and *S. aureus*.

**TABLE 4:** Cumulative fraction of response for *Streptococcus pneumoniae* and *Staphylococcus aureus* in plasma and epithelial lining fluid with original and higher plasma protein binding after lefamulin intravenous and oral administration.

Dosage regimen			*Streptococcus pneumoniae*		*Staphylococcus aureus*	
			1-log	2-log	1-log	2-log
Original ppb	150 mg iv 1/1.5/2 h	Plasma	100	100	100	100
		ELF	100	100	100	100
	600 mg oral fasted	Plasma	100	100	100	100
		ELF	100	100	100	99
	600 mg oral fed	Plasma	100	100	100	100
		ELF	100	100	100	98
Higher ppb	150 mg iv 1/1.5/2 h	Plasma	100	99	100	97
		ELF	100	100	100	99
	600 mg oral fasted	Plasma	99	97	100	94
		ELF	100	100	100	100
	600 mg oral fed	Plasma	98	93	99	88
		ELF	100	100	100	99

Original ppb refers to the diluted plasma protein binding, higher ppb refers to the non-diluted plasma protein binding rate. ELF, epithelial lining fluid.

## Discussion

Lefamulin is a potential antimicrobial agent for CABP caused by *S. pneumoniae* and *S. aureus* (Veve and Wagner, 2018; Eraikhuemen et al., 2021). This study evaluated the PTA and CFR for different administration regimens of intravenous infusion of lefamulin 150 mg q12 h over 1 h, 1.5 h, extending the infusion to 2 h, and oral administration of lefamulin 600 mg q12 h under fasted or fed conditions in the treatment of Chinese patients with CABP. The study also assessed the impact of original and higher plasma protein binding rates on microbiological efficacy.

This study included 183 blood concentration data points from 33 Chinese CABP patients as a validation dataset, validating the lefamulin PopPK model published by the FDA (9). The results indicated that the distribution characteristics of drug concentration-time curves in Chinese patients with CABP were similar to those observed in foreign clinical studies. The validation of the established foreign lefamulin PopPK model using clinical study data from Chinese CABP patients demonstrated acceptable accuracy and precision. Therefore, this study employed the PopPK model to simulate the exposure levels in the plasma and epithelial lining fluid (ELF) of Chinese CABP patients under different dosing regimens and conducted PK/PD analysis.

Foreign pharmacodynamic data indicate that lefamulin has MIC_90_ values of 0.12 mg/L for both *S. pneumoniae* and *S. aureus* (Paukner et al., 2019). The corresponding MIC_90_ values for Chinese clinically isolated strains are 0.125 and 0.06 mg/L, respectively, similar to those observed in foreign studies (Wu et al., 2020). Foreign PK/PD analysis demonstrates that, under the regimen of lefamulin 150 mg q12 h infused over 1 h, the probability of achieving the pharmacodynamic target of a 1-log_10_ CFU/mL reduction for *S. pneumoniae* and *S. aureus* is above 99% at the MIC_90_ of 0.12 mg/L in both plasma and epithelial lining fluid (ELF) (Bhavnani et al., 2019; Bhavnani and Rubino, 1976).

Our results indicate that extending the infusion time has no significant impact on the exposure levels and PTA for lefamulin in both plasma and ELF. It is recommended to use an extended infusion method to mitigate adverse reactions at the infusion site. From clinical aspect, in the Phase III Lefamulin Evaluation Against Pneumonia (LEAP 1) Trial, lefamulin (150 mg q12 h IV followed by 600 mg q12 h orally) was noninferior to moxifloxacin (400 mg qd IV followed by 400 mg qd orally) for early clinical response (87.3% vs. 90.2%, respectively) and investigator assessment of clinical response (mITT, 81.7% vs. 84.2%, respectively; CE, 86.9% vs. 89.4%, respectively). In microbiological ITT population, lefamulin produced 88.2% and 100% early clinical response agaisnt *S. pneumoniae* and *S. aureus*. In the Chinese bridging trial, the investigator assessment of clinical response was 76.8% in the lefamulin group and 71.4% in the moxifloxacin group. The above clinical efficacy results demonstrated the effectiveness of the recommended regimen based on PK/PD analysis. There was a slight decrease of drug exposure in plasma and ELF in the fed state, which was in accordance with the slightly reduced bioavailability in subjects who consumed high-fat meals (Wicha et al., 2019b). It was reported that the absolute oral bioavailability is 25.8% in the fasted state and 21.0% in the fed state (Wicha et al., 2017). However, regardless of fasted or fed conditions, the PTA for oral administration of lefamulin 600 mg q12 h is comparable to the intravenous infusion of 150 mg q12 h, supporting the sequential intravenous and oral treatment of CABP patients caused by *S. pneumoniae* and *S. aureus* (Xenleta, 2019).

This study conducted PK/PD analysis based on the exposure levels of plasma-free drug and ELF drug at steady state under different protein binding rates. At the MIC_90_ values for *S. pneumoniae* and *S. aureus*, the probability of achieving the pharmacodynamic target of a 1-log_10_ CFU/mL reduction in plasma remains above 98%. High protein binding had no significant impact on ELF drug exposure levels and the probability of reaching the target, with the highest MIC covered by PTA at 90% being essentially the same for *S. pneumoniae* and *S. aureus* using original and higher protein binding. The above results indicate that PK/PD assessment should be conducted according to drug exposure at the infection site. More attention should be given to the drug concentrations at the site of infection, considering the influence of protein binding.

This study provides data support for the selection of lefamulin 150 mg q12 h with extended infusion from 1 h to 2 h and oral administration of 600 mg q12 h with or without food for the treatment of Chinese CABP. It is anticipated that the aforementioned dosing regimens will exhibit good microbiological efficacy against CABP caused by *S. pneumoniae* and *S. aureus*.

Several limitations of this study should be mentioned here. In the PK prediction for intravenous administration, MAPE and RMSE are slightly higher. Further model refinement should be done to improve the predictive accuracy. Additionally, future studies incorporating real-world data are necessary to further validate these conclusions.

## Data Availability

The data that support the findings of this study are available from the corresponding author upon reasonable request.
