# Supplementary material for: Lefamulin dosing optimization using population pharmacokinetic and pharmacokinetic/pharmacodynamic assessment in Chinese patients with community-acquired bacterial pneumonia
Source: Front Pharmacol. 2024 Oct 25;15:1456741. doi: 10.3389/fphar.2024.1456741 (PMC11544005; doi:10.3389/fphar.2024.1456741)
Supplement: Supplementary file 1 [file DataSheet1.docx]

**Table S1** Parameter list for the lefamulin final PopPK model with original plasma protein binding

| **Typical value** |  |  |
| --- | --- | --- |
| **Theta (θ)** | **Description (Units)** | **Final Estimate** |
| 1 | CL (L/hr) | 79.4 |
| 2 | Vc (L) | 46.3 |
| 3 | CLd1 (L/hr) | 40.6 |
| 4 | Vp1 (L) | 249 |
| 5 | CLd2 (L/hr) | 199 |
| 6 | Vp2 (L) | 259 |
| 7 | Ka (hr^-1^) | 1.2 |
| 8 | Ka2 (hr^-1^) | 2.12 |
| 9 | Ftot | 0.244 |
| 10 | FS | 0.802 |
| 11 | ALAG (hr) | 0.15 |
| 12 | fu, min | 0.0997 Fixed |
| 13 | fu, max | 0.259 Fixed |
| 14 | fu50 (mg/L) | 1.35 Fixed |
| 17 | Ka2fed (1/hr) | 0.445 |
| 18 | Ftot, fed | 0.763 |
| 19 | Kafed (1/hr) | 0.0541 |
| 20 | The effect of albumin on CL | 1.214 |
| 22 | The effect of Phase 1 on CL | 1.766 |
| 21 | The effect of Phase 2 on CL | 1.827 |
| 24 | The effect of Phase 1 on CLd1 | 2.12 |
| 23 | The effect of Phase 2 on CLd1 | 1.44 |
| 27 | The effect of WTKG on Vp1 | 1.0129 |
| 26 | The effect of Phase 1 on Vp1 | 2.75 |
| 25 | The effect of Phase 2 on Vp1 | 1.985 |
| 28 | KELF, in | 2.71 |
| 29 | KELF, out | 0.51 |
| **Interindividual variability** |  |  |
| **Eta (η)** | **Description** | **Final Estimate** |
| 1 | ω^2^CL | 0.171 (41.4%CV) |
| 2 | ω^2^V_c_ | 0.39 (62.4%CV) |
| 3 | ω^2^CLd_1_ | 0.119 (34.5%CV) |
| 4 | ω^2^V_p1_ | 0.623 (78.9%CV) |
| 7 | ω^2^K_a_ | 0.800 (89.4%CV) |
| 8 | ω^2^K_a2_ | 0.400 (63.2%CV) |
| 9 | ω^2^F_tot_ | 0.100 (31.6%CV) |
| 10 | ω^2^FS | 0.170 (41.2%CV) |
| **Residual variability** |  |  |
| **Epsilon (ε)** | **Description** | **Final Estimate** |
| 1 | σ^2^Proportional | 0.103 (32.0%CV) |
| 2 | σ^2^Additive | 0.0000343 (0.00586 mg/L) |
| 3 | σ^2^Proportional | 0.372 (61.0%CV) |

**Table S2** Parameter list for the lefamulin final PopPK model with higher plasma protein binding

| **Typical value** |  |  |
| --- | --- | --- |
| **Theta (θ)** | **Description (Units)** | **Final Estimate** |
| 1 | CL (L/hr) | 282 |
| 2 | Vc (L) | 138 |
| 3 | CLd1 (L/hr) | 187 |
| 4 | Vp1 (L) | 1300 |
| 5 | CLd2 (L/hr) | 421 |
| 6 | Vp2 (L) | 449 |
| 7 | Ka (hr-1) | 0.686 |
| 8 | Ka2 (hr-1) | 1.34 |
| 9 | Ftot | 0.293 |
| 10 | FS | 0.622 |
| 11 | ALAG (hr) | 0.124 |
| 12 | fu, min | 0.0260 Fixed |
| 13 | fu, max | 0.194 Fixed |
| 14 | fu50 (mg/L) | 0.814 Fixed |
| 17 | Ka2fed (1/hr) | 0.513 |
| 18 | Ftot, fed | 0.802 |
| 19 | Kafed (1/hr) | 0.0525 |
| 20 | The effect of albumin on CL | 1.2 |
| 22 | The effect of Phase 1 on CL | 1.710 |
| 21 | The effect of Phase 2 on CL | 1.707 |
| 24 | The effect of Phase 1 on CLd1 | 1.788 |
| 23 | The effect of Phase 2 on CLd1 | 1.192 |
| 27 | The effect of WTKG on Vp1 | 1.00637 |
| 26 | The effect of Phase 1 on Vp1 | 1.889 |
| 25 | The effect of Phase 2 on Vp1 | 1.28 |
| 28 | LPR | 2.71 |
| 29 | Power | 0.51 |
| **Interindividual variability** |  |  |
| **Eta (η)** | **Description** | **Final Estimate** |
| 1 | ω^2^CL | 0.151 (38.9%CV) |
| 2 | ω^2^V_c_ | 0.195 (44.2%CV) |
| 3 | ω^2^CLd_1_ | 0.064 (25.3%CV) |
| 4 | ω^2^V_p1_ | 0.0875 (29.6%CV) |
| 7 | ω^2^K_a_ | 2.65 (162.8%CV) |
| 8 | ω^2^K_a2_ | 0.264 (51.4%CV) |
| 9 | ω^2^F_tot_ | 0.791 (88.9%CV) |
| 10 | ω^2^FS | 0.716 (87.2%CV) |
| **Residual variability** |  |  |
| **Epsilon (ε)** | **Description** | **Final Estimate** |
| 1 | σ^2^Proportional | 0.0958 (31.0%CV) |
| 2 | σ^2^Additive | 0.0000167 (0. 0000167 mg/L) |
| 3 | σ^2^Proportional | 0.192 (43.8%CV) |

**Table S3** MIC distributions of lefamulin to *Streptococcus pneumoniae and Staphylococcus aureus*

| **Bacteria (number)** | **MIC distribution frequency** | | | | | **MIC_50_/MIC_90_（mg/L）** |
| --- | --- | --- | --- | --- | --- | --- |
|  | 0.015 | 0.03 | 0.06 | 0.125 | 0.25 |  |
| *Streptococcus pneumoniae* (172) | 2.91 | 6.98 | 21.51 | 59.3 | 9.3 | 0.125/0.125 |
| *Staphylococcus aureus* (121) | 38.84 | 2.48 | 50.41 | 7.44 | 0.83 | 0.06/0.06 |

MIC_50_/MIC_90_ represent the minimum concentrations that can inhibit 50%/90% bacteria growth

**Table S4** PK/PD targets of lefamulin to *Streptococcus pneumoniae and Staphylococcus aureus*

| **Bacteria** | **Δlog_10_ CFU= -1** | | **Δlog_10_ CFU= -2** | | |  |
| --- | --- | --- | --- | --- | --- | --- |
|  | ***f*AUC_0-24h, plasma_/MIC** | **AUC_0-24h,ELF_/MIC** | | ***f*AUC_0-24h, plasma_/MIC** | **AUC_0-24h,ELF_/MIC** |  |
| *Streptococcus pneumoniae* | 1.37 | 14.0 | | 2.15 | 22 |  |
| *Staphylococcus aureus* | 2.13 | 21.7 | | 6.24 | 63.9 |  |

*f*AUC_0-24h,plasma_ represents the free drug exposure from 0-24 h at steady state，AUC_0-24h,ELF_ represents the total drug exposure from 0-24 h at steady state. The PK/PD targets were set based on pharmacodynamic response of five *Streptococcus pneumoniae and Staphylococcus aureus* in neutropenic mouse lung infection model.

**Table S5** Summary of demographic information for modeling dataset and external validation (Phase III clinical trial in China) dataset.

|  | **Model building population** | | | | **External validation population** |
| --- | --- | --- | --- | --- | --- |
| **Variable** | **Phase 1**  **(N = 98)**  **Median**  **(Min.-Max.)** | **Phase 2**  **(N = 129)**  **Median**  **(Min.-Max.)** | **Phase 3**  **(N = 622)**  **Median**  **(Min.-Max.)** | **Total**  **(N = 849)**  **Median**  **(Min.-Max.)** | **Phase 3**  **(N = 33)**  **Median**  **(Min.-Max.)** |
| Age (yr) | 50 | 41 | 61 | 57 | 56.12 |
|  | (19-77) | (18-73) | (19-97) | (18-97) | (26-78) |
| Height (cm) | 173 | 173 | 168 | 170 | 169.7 |
|  | (146-191) | (150-196) | (133-200) | (133-200) | (155-180) |
| Weight (kg) | 83.4 | 87.5 | 75 | 78 | 69.38 |
|  | (54-124) | (43.8-161) | (31-175) | (31-174.6) | (40-112) |
| BSA (m^2^) | 1.99 | 2.02 | 1.85 | 1.89 | 1.79 |
|  | (1.53-2.44) | (1.4-2.68) | (1.13-2.73) | (1.13-2.73) | (1.37-2.3) |
| BMI (kg/m^2^) | 27.6 | 30.3 | 26 | 26.58 | 24.00 |
|  | (19.7-38.5) | (12.1-55.5) | (13-56.8) | (12.1-56.82) | (15.63-34.57) |
| CLCR (mL/min) | 95.8 | 106 | 72.5 | 80.2 | 92.51 |
|  | (6.2-149) | (25.6-191) | (15.2-192) | (6.2-192.4) | (37.72-139.04) |
| CLCRN (mL/min/1.73 m^2^) | 87.8 | 87.6 | 68.8 | 73.4 | 88.99 |
|  | (5.4-130) | (24.1-171) | (14.1-192) | (5.4-192.4) | (47.63-132.47) |
| Albumin (g/dL) | 4.5 | 4.2 | 4 | 4.1 | 3.88 |
|  | (2.8-5.6) | (2.8-5.2) | (2-5.3) | (2-5.6) | (2.9-4.8) |
| Gender |  |  |  |  |  |
| Male | 74/98 (75.5%) | 86/129 (66.7%) | 360/622 (57.9%) | 520/849 (61.2%) | 27/33  (81.82%) |
| Female | 24/98 (24.5%) | 43/129 (33.3%) | 262/622 (42.1%) | 329/849 (38.8%) | 6/33  (18.18%) |
| Race |  |  |  |  |  |
| White | 74/98 (75.5%) | 97/129 (75.2%) | 493/622 (79.3%) | 664/849 (78.2%) |  |
| Black | 20/98 (20.4%) | 20/129 (15.5%) | 29/622 (4.66%) | 69/849 (8.13%) |  |
| Asian | 1/98  (1.02%) | 1/129 (0.775%) | 70/622 (11.3%) | 72/849 (8.48%) | 33/33  (100%) |
| American Indian/ Alaskan Native | 2/98  (2.04%) | 6/129 (4.65%) | 23/622  (3.7%) | 31/849 (3.65%) |  |
| Native-Hawaiian/  Other Pacific Islander | - | 4/129  (3.1%) | - | 4/849 (0.471%) |  |
| Other | 1/98  (1.02%) | 1/129 (0.775%) | 7/622  (1.13%) | 9/849  (1.06%) |  |

**Table S6** Exposure comparisons between plasma free-drug and ELF drug with original and higher plasma protein binding rates

|  | **Exposure of free drug in plasma (mg*h/L)** | | | | | | | | | |
| --- | --- | --- | --- | --- | --- | --- | --- | --- | --- | --- |
|  | **150 mg q12h iv 1 h** | | **150 mg q12h iv 1.5 h** | | **150 mg q12h iv 2 h** | | **600 mg q12h fasted** | | **600 mg q12h fed** | |
|  | **Day 1** | **Day 3** | **Day 1** | **Day 3** | **Day 1** | **Day 3** | **Day 1** | **Day 3** | **Day 1** | **Day 3** |
| **Original ppb** | 3.25  (0.81,9.36) | 3.87  (0.87,19.01) | 3.24  (0.81,9.27) | 3.86  (0.87,18.99) | 3.23  (0.81,9.17) | 3.86  (0.87,18.97) | 3.02  (0.61,10.00) | 3.71  (0.66,21.59) | 1.89  (0.22,7.29) | 2.76  (0.48,16.28) |
| **Higher ppb** | 0.92  (0.25,2.32) | 1.08  (0.25,3.65) | 0.92  (0.24,2.30) | 1.08  (0.25,3.65) | 0.92  (0.24,2.29) | 1.08  (0.25,3.64) | 0.98  (0.04,5.27) | 1.26  (0.06,8.68) | 0.57  (0.02,4.23) | 0.89  (0.03,7.81) |
|  | **Exposure of drug in ELF (mg*h/L)** | | | | | | | | | |
|  | **150 mg q12h iv 1 h** | | **150 mg q12h iv 1.5 h** | | **150 mg q12h iv 2 h** | | **600 mg q12h fasted** | | **600 mg q12h fed** | |
|  | **Day 1** | **Day 3** | **Day 1** | **Day 3** | **Day 1** | **Day 3** | **Day 1** | **Day 3** | **Day 1** | **Day 3** |
| **Original ppb** | 16.64 (4.23,45.58) | 20.51 (4.61,100.02) | 16.55 (4.22,45.03) | 20.51 (4.61,99.89) | 16.45 (4.21,44.50) | 20.50 (4.61,99.75) | 15.22 (3.17,49.06) | 19.69 (3.5,113.13) | 9.22 (0.98,35.05) | 14.64 (2.55,85.17) |
| **Higher ppb** | 14.75 (6.50,21.94) | 16.12 (6.57,25.35) | 14.96 (6.84,22.02) | 16.32 (6.91,25.39) | 15.15 (7.13,22.06) | 16.48 (7.20,25.42) | 16.36 (4.14,27.69) | 18.10 (5.04,31.88) | 13.45 (2.62,25.71) | 16.49 (3.54,31.07) |

Results were shown as median (minimum, maximum), original ppb refers to the diluted plasma protein binding, higher ppb refers to the non-diluted plasma protein binding rate. ELF, epithelial lining fluid.

**Table S7** Probability of target attainment for *Streptococcus pneumoniae* and *Staphylococcus aureus* in plasma and epithelial lining fluid with original and higher plasma protein binding after lefamulin intravenous and oral administration

A. Original ppb

| **Dosage regimen** | ***S. pneumoniae* (MIC_90_=0.125 mg/L)** | **PK/PD target** | **PTA (%)** | | | | | | |
| --- | --- | --- | --- | --- | --- | --- | --- | --- | --- |
|  |  |  | **0.015** | **0.03** | **0.06** | **0.125** | **0.25** | **0.5** | **1** |
| 150 mg iv 1/1.5/2 h | Plasma | 1.37 | 100 | 100 | 100 | 100 | 100 | 100 | 99 |
|  |  | 2.15 | 100 | 100 | 100 | 100 | 100 | 100 | 88 |
|  | ELF | 14 | 100 | 100 | 100 | 100 | 100 | 99 | 77 |
|  |  | 22 | 100 | 100 | 100 | 100 | 100 | 90 | 39 |
| 600 mg oral fasted | Plasma | 1.37 | 100 | 100 | 100 | 100 | 100 | 100 | 98 |
|  |  | 2.15 | 100 | 100 | 100 | 100 | 100 | 100 | 87 |
|  | ELF | 14 | 100 | 100 | 100 | 100 | 100 | 98 | 75 |
|  |  | 22 | 100 | 100 | 100 | 100 | 100 | 88 | 40 |
| 600 mg oral fed | Plasma | 1.37 | 100 | 100 | 100 | 100 | 100 | 100 | 92 |
|  |  | 2.15 | 100 | 100 | 100 | 100 | 100 | 97 | 69 |
|  | ELF | 14 | 100 | 100 | 100 | 100 | 100 | 93 | 52 |
|  |  | 22 | 100 | 100 | 100 | 100 | 98 | 71 | 18 |
| **Dosage regimen** | ***S. aureus***  **(MIC_90_=0.06 mg/L)** | **PK/PD target** | **PTA (%)** | | | | | | |
|  |  |  | **0.015** | **0.03** | **0.06** | **0.125** | **0.25** | **0.5** | **1** |
| 150 mg iv 1/1.5/2 h | Plasma | 2.13 | 100 | 100 | 100 | 100 | 100 | 100 | 89 |
|  |  | 6.24 | 100 | 100 | 100 | 100 | 97 | 64 | 11 |
|  | ELF | 21.7 | 100 | 100 | 100 | 100 | 100 | 90 | 40 |
|  |  | 63.9 | 100 | 100 | 100 | 98 | 67 | 13 | 1 |
| 600 mg oral fasted | Plasma | 2.13 | 100 | 100 | 100 | 100 | 100 | 100 | 88 |
|  |  | 6.24 | 100 | 100 | 100 | 100 | 97 | 64 | 13 |
|  | ELF | 21.7 | 100 | 100 | 100 | 100 | 100 | 89 | 41 |
|  |  | 63.9 | 100 | 100 | 100 | 97 | 65 | 15 | 1 |
| 600 mg oral fed | Plasma | 2.13 | 100 | 100 | 100 | 100 | 100 | 97 | 70 |
|  |  | 6.24 | 100 | 100 | 100 | 100 | 88 | 40 | 5 |
|  | ELF | 21.7 | 100 | 100 | 100 | 100 | 98 | 72 | 19 |
|  |  | 63.9 | 100 | 100 | 100 | 88 | 42 | 5 | 0 |

B. Higher ppb

| **Dosage regimen** | ***S. pneumoniae* (MIC_90_=0.125 mg/L)** | **PK/PD target** | **PTA (%)** | | | | | |
| --- | --- | --- | --- | --- | --- | --- | --- | --- |
|  |  |  | **0.015** | **0.03** | **0.06** | **0.125** | **0.25** | **0.5** |
| 150 mg iv 1/1.5/2 h | Plasma | 1.37 | 100 | 100 | 100 | 100 | 100 | 84 |
|  |  | 2.15 | 100 | 100 | 100 | 100 | 94 | 48 |
|  | ELF | 14 | 100 | 100 | 100 | 100 | 100 | 100 |
|  |  | 22 | 100 | 100 | 100 | 100 | 100 | 94 |
| 600 mg oral fasted | Plasma | 1.37 | 100 | 100 | 100 | 100 | 95 | 76 |
|  |  | 2.15 | 100 | 100 | 100 | 98 | 85 | 54 |
|  | ELF | 14 | 100 | 100 | 100 | 100 | 100 | 100 |
|  |  | 22 | 100 | 100 | 100 | 100 | 100 | 95 |
| 600 mg oral fed | Plasma | 1.37 | 100 | 100 | 100 | 98 | 87 | 61 |
|  |  | 2.15 | 100 | 100 | 99 | 93 | 72 | 39 |
|  | ELF | 14 | 100 | 100 | 100 | 100 | 100 | 100 |
|  |  | 22 | 100 | 100 | 100 | 100 | 100 | 88 |
| **Dosage regimen** | ***S. aureus***  **(MIC_90_=0.06 mg/L)** | **PK/PD target** | **PTA (%)** | | | | | |
|  |  |  | **0.015** | **0.03** | **0.06** | **0.125** | **0.25** | **0.5** |
| 150 mg iv 1/1.5/2 h | Plasma | 2.13 | 100 | 100 | 100 | 100 | 94 | 49 |
|  |  | 6.24 | 100 | 100 | 99 | 76 | 17 | 1 |
|  | ELF | 21.7 | 100 | 100 | 100 | 100 | 100 | 95 |
|  |  | 63.9 | 100 | 100 | 100 | 100 | 45 | 0 |
| 600 mg oral fasted | Plasma | 2.13 | 100 | 100 | 100 | 98 | 85 | 55 |
|  |  | 6.24 | 100 | 99 | 94 | 70 | 34 | 9 |
|  | ELF | 21.7 | 100 | 100 | 100 | 100 | 100 | 96 |
|  |  | 63.9 | 100 | 100 | 100 | 100 | 61 | 1 |
| 600 mg oral fed | Plasma | 2.13 | 100 | 100 | 99 | 93 | 73 | 39 |
|  |  | 6.24 | 100 | 97 | 85 | 55 | 22 | 5 |
|  | ELF | 21.7 | 100 | 100 | 100 | 100 | 100 | 89 |
|  |  | 63.9 | 100 | 100 | 100 | 99 | 48 | 1 |

PK/PD targets corresponding to 1-log_10_/2-log_10_CFU/mL bacteria reductions were utilized. Original ppb refers to the diluted plasma protein binding, higher ppb refers to the non-diluted plasma protein binding rate. *S. pneumoniae, Streptococcus pneumoniae, S. aureus, Staphylococcus aureus,* PTA, probability of target attainment, ELF, epithelial lining fluid.

**Table S8** Pharmacokinetic/pharmacodynamic breakpoints for *Streptococcus pneumoniae* and *Staphylococcus aureus* in plasma and epithelial lining fluid with original and higher plasma protein binding after lefamulin intravenous and oral administration

| **Dosage regimen** |  | |  | | ***S. pneumoniae*** | | | ***S. aureus*** | | | |
| --- | --- | --- | --- | --- | --- | --- | --- | --- | --- | --- | --- |
|  |  |  | **Plasma** | | | **ELF** | | **Plasma** | | **ELF** | |
|  | |  | 1-log | 2-log | | 1-log | 2-log | 1-log | 2-log | 1-log | 2-log |
| 150 mg iv 1/1.5/2 h | | Original ppb | 1 | 0.5 | | 0.5 | 0.5 | 0.5 | 0.25 | 0.5 | 0.125 |
|  |  | Higher ppb | 0.25 | 0.25 | | 0.5 | 0.5 | 0.25 | 0.06 | 0.5 | 0.125 |
| 600 mg oral fasted | | Original ppb | 1 | 0.5 | | 0.5 | 0.25 | 0.5 | 0.25 | 0.25 | 0.125 |
|  |  | Higher ppb | 0.25 | 0.125 | | 0.5 | 0.5 | 0.125 | 0.06 | 0.5 | 0.125 |
| 600 mg oral fed | | Original ppb | 1 | 0.5 | | 0.5 | 0.25 | 0.5 | 0.125 | 0.25 | 0.06 |
|  |  | Higher ppb | 0.125 | 0.125 | | 0.5 | 0.25 | 0.125 | 0.03 | 0.25 | 0.125 |
| MIC_50_ (mg/L) | | | 0.125 | | | | | 0.06 | | | |
| MIC_90_ (mg/L) | | | 0.125 | | | | | 0.06 | | | |

The pharmacokinetic/pharmacodynamic breakpoints were based on the exposure levels achieved at steady state with the dosing regimen of lefamulin 150 mg q12h infused over 1/1.5/2 h or 600 mg oral administrations. PK/PD targets corresponding to 1-log_10_/2-log_10_CFU/mL bacteria reductions were utilized. Original ppb refers to the diluted plasma protein binding, higher ppb refers to the non-diluted plasma protein binding rate. ELF, epithelial lining fluid, *S. pneumoniae*, *Streptococcus pneumoniae*, *S. aureus, Staphylococcus aureus*.


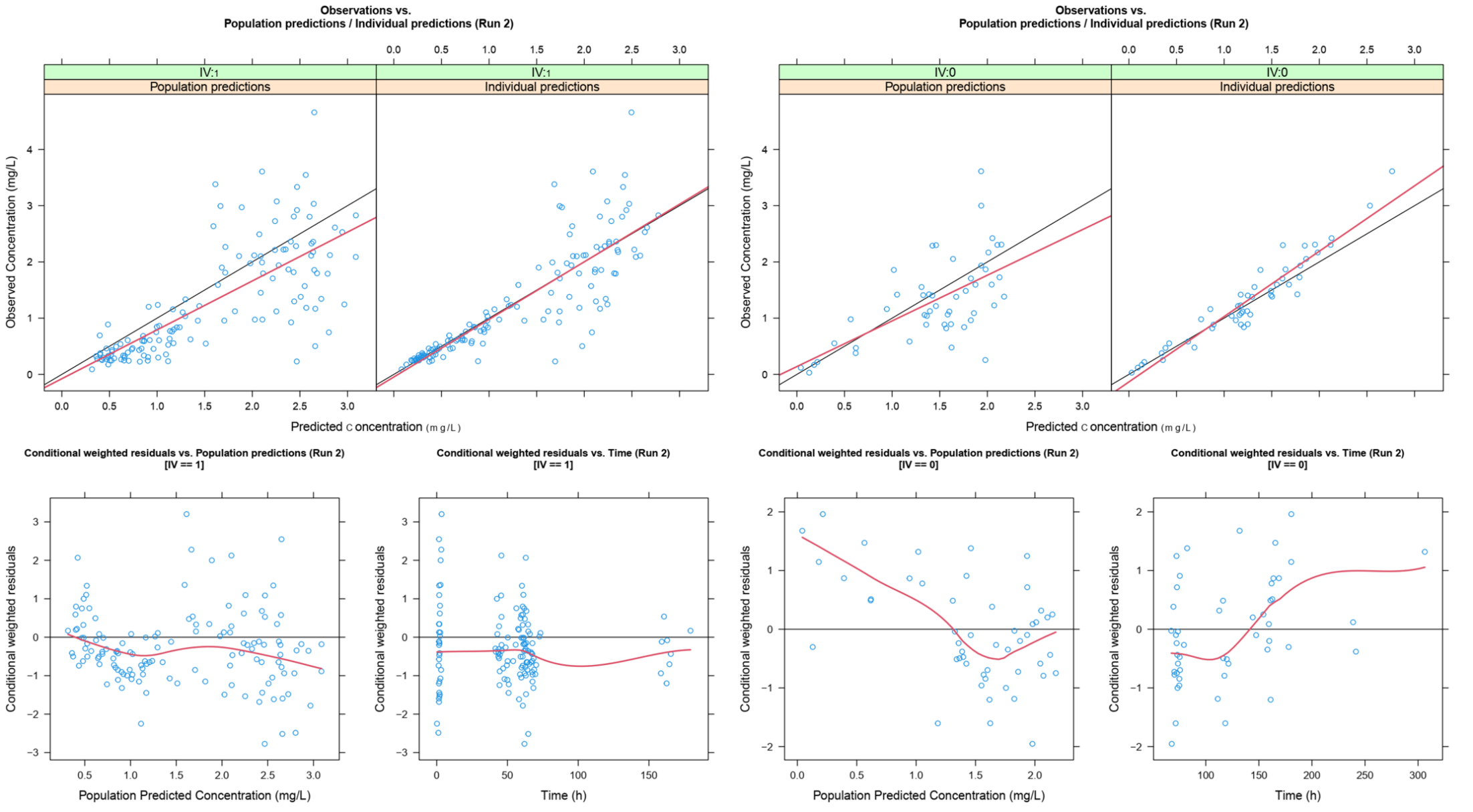


**Fig. S1** Goodness-of-fit (GOF) plots for the final lefamulin PopPK model with different administration routes.

Left plot represents the GOF plot for intravenous administration, and Right plot represents the GOF plot for oral administration. The upper-left plot is the GOF plot of observed values versus population predictions, the upper-right plot is the GOF plot of observed values versus individual predictions, the lower-left plot is the GOF plot of conditional weighted residuals versus population predictions, and the lower-right plot is the GOF plot of conditional weighted residuals versus time. Blue dots represent observed values, the solid black line represents the diagonal line, and the red line represents the loess regression curve.


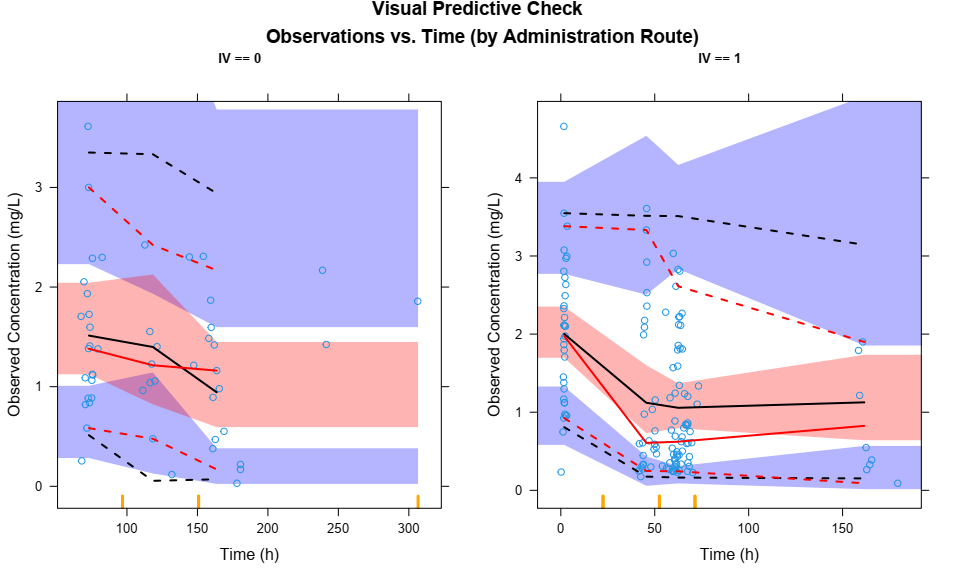


**Fig. S2** Visual Predictive Check (VPC) plots for the final lefamulin PopPK model with different administration routes.

Left plot represents the VPC plot for oral administration, and Right plot represents the VPC plot for intravenous administration. The solid red line in the plot indicates the 50th percentile of observed values, and the red dashed lines represent the 95th and 5th percentiles of observed values, respectively. The solid black line represents the 50th percentile of predicted values, and the black dashed lines represent the 95th and 5th percentiles of predicted values, respectively. The blue and red shaded areas represent the 90% confidence intervals for the corresponding predicted percentiles.


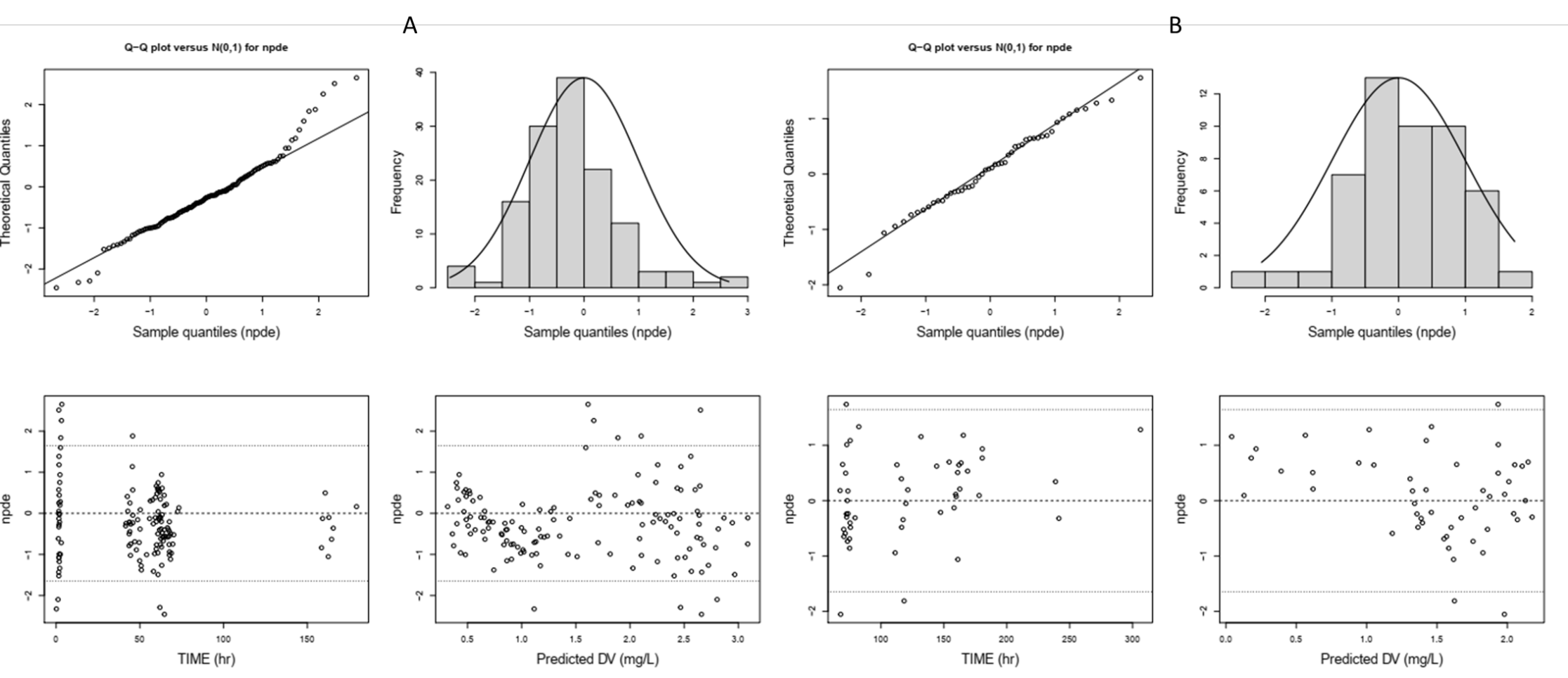


**Fig. S3** Normalized Prediction Distribution Errors (NPDE) distribution plots for the final lefamulin PopPK model with different administration routes.

(A) represents the NPDE distribution plot for intravenous administration, and (B) represents the NPDE distribution plot for oral administration. From the top-left to the bottom-right, the plots include NPDE-standard normal distribution QQ plot, NPDE histogram, NPDE versus time plot, and NPDE versus predicted concentration plot.

**Fig. S4** Exposure comparisons between plasma free-drug and ELF drug with original and higher plasma protein binding rates.

The box plots show the maximum, minimum and median values. Original ppb refers to the diluted plasma protein binding, higher ppb refers to the non-diluted plasma protein binding rate. ELF, epithelial lining fluid. *f*AUC_0-24h,plasma_, the area under the curve of plasma-free drug concentration over time from time of dosing to 24 h, AUC_0-24h,ELF,_ the area under the curve of ELF drug concentration over time from time of dosing to 24 h.
